# The Call

**Published:** 1866-06

**Authors:** 


### Editorial.

### THE CALL.

The following call issued to the dentists of Ohio we regard as a very important step and one that should attract the attention of the entire profession of the State. We hope and confidently expect to meet a large number of our professional brethren on that occasion. It will be the largest dental meeting ever held in the state; and it is important it should be; every state in the Union should have its State Dental Association.

In this organization it is very important that all who have the welfare and best interests of the profession at heart should participate.

There are in the state over five hundred dentists, and we have good reason to believe that over half that number will be present. We do most sincerely hope that the formation of this society will be accomplished, by the harmonious cooperation of the entire profession of the State. Let every man be a committee, to see to it that he himself is promptly there on the morning of the 26th inst that the meeting may be called together at 10 o'clock A. M. Let no one harbor the thought for one moment, that it will make no difference if he is not there promptly. It will make a difference, and perhaps a fatal one. So let all be there, and every one take somebody with him.

